# Fiber-specific white matter reductions in Parkinson hallucinations and visual dysfunction

**DOI:** 10.1212/WNL.0000000000009014

**Published:** 2020-04-07

**Authors:** Angeliki Zarkali, Peter McColgan, Louise-Ann Leyland, Andrew J. Lees, Geraint Rees, Rimona S. Weil

**Affiliations:** From the Dementia Research Centre (A.Z., L.-A.L., R.S.W.), Huntington's Disease Centre (P.M.), Institute of Cognitive Neuroscience (G.R.), and Wellcome Centre for Human Neuroimaging (G.R., R.S.W.), University College London; and Reta Lila Weston Institute of Neurological Studies (A.J.L.), London, UK.

## Abstract

**Objective:**

To investigate the microstructural and macrostructural white matter changes that accompany visual hallucinations and low visual performance in Parkinson disease, a risk factor for Parkinson dementia.

**Methods:**

We performed fixel-based analysis, a novel technique that provides metrics of specific fiber-bundle populations within a voxel (or fixel). Diffusion MRI data were acquired from patients with Parkinson disease (n = 105, of whom 34 were low visual performers and 19 were hallucinators) and age-matched controls (n = 35). We used whole-brain fixel-based analysis to compare microstructural differences in fiber density (FD), macrostructural differences in fiber bundle cross section (FC), and the combined FD and FC (FDC) metric across all white matter fixels. We then performed a tract-of-interest analysis comparing the most sensitive FDC metric across 11 tracts within the visual system.

**Results:**

Patients with Parkinson disease hallucinations exhibited macrostructural changes (reduced FC) within the splenium of the corpus callosum and the left posterior thalamic radiation compared to patients without hallucinations. While there were no significant changes in FD, we found large reductions in the combined FDC metric in Parkinson hallucinators within the splenium (>50% reduction compared to nonhallucinators). Patients with Parkinson disease and low visual performance showed widespread microstructural and macrostructural changes within the genu and splenium of the corpus callosum, bilateral posterior thalamic radiations, and left inferior fronto-occipital fasciculus.

**Conclusions:**

We demonstrate specific white matter tract degeneration affecting posterior thalamic tracts in patients with Parkinson disease with hallucinations and low visual performance, providing direct mechanistic support for attentional models of visual hallucinations.

Dementia is common in PD over the age of 65 years,^1^ but it is impossible to determine which patients will develop cognitive impairment at the time of diagnosis.^2,3^ One recognized risk factor is the occurrence of visual hallucinations (VH).^4^ Similarly, visual processing abnormalities are associated with higher risk: poor color vision is linked to Parkinson dementia,^5^ and impaired visual performance correlates with poor cognition at the 1-year follow-up.^6,7^ The neural substrates of hallucinations and visual dysfunction therefore provide important insights into early Parkinson dementia.

Axonal involvement occurs early in PD. Dystrophic axonal changes precede neuronal loss^8^; exogenous α-synuclein in neuronal cultures triggers endogenous pathology within axons^9^; and in mouse mutant models, axonal pathology develops before neurite loss.^10^ Techniques sensitive to axonal changes such as diffusion-weighted imaging (DWI) are most likely to detect early cognitive involvement in PD. DWI studies show white matter changes in patients with PD without dementia without gray matter atrophy^11,12^; these are worse in patients with poorer cognition^11,12^ and established dementia.^13,14^ However, the imaging changes accompanying or preceding the earliest stages of Parkinson dementia are not fully established.

Fixel-based analysis is a novel framework providing metrics particularly sensitive to white matter microstructural and macrostructural changes. We used this technique to measure fiber-specific changes in patients with PD vs controls, PD/VH vs PD/non-VH, and patients with PD with poor visual performance vs those with PD with maintained performance. We hypothesized that white matter atrophy would be present in patients with PD with hallucinations and visual dysfunction, who are at risk of dementia.

## Methods

### Participants

We recruited 105 patients with PD to our UK center. All patients satisfied the Queen Square Brain Bank criteria for PD.^15^ Thirty-five unaffected controls were recruited from spouses and volunteer databases.

Patients with PD were classified as habitual visual hallucinators (PD/VH) if they scored ≥1 on question 2 of the Movement Disorder Society Unified Parkinson's Disease Rating Scale (UPDRS; “Over the past week have you seen, heard, smelled or felt things that were not really there?”). Nineteen patients answered positively to this question (PD/VH) and 86 did not (PD/non-VH). Further details on the experienced hallucinatory phenomena were collected with the University of Miami Parkinson's Disease Hallucinations Questionnaire, which quantifies hallucination severity and frequency (maximum score 14).

The patients with PD were also classified into high visual performers (n = 70) and low visual performers (n = 34) on the basis of performance on 2 visual computer tasks (Cats-and-Dogs task and Biological Motion task). Details of stimulus generation and experimental procedures have been described previously^6,7,16^ (example stimuli are available from Figshare, supplemental figure 1, figshare.com/s/1ff87201e92924f488f2). For both tasks, performance was measured in terms of discrimination sensitivity; participants who performed worse than the group median performance on both tasks were classified as low visual performers. One participant with PD was unable to successfully complete both visual tasks and was excluded from analysis.

### Standard protocol approvals, registrations, and patient consents

The study was approved by the Queen Square ethics committee, and all participants provided written informed consent before taking part.

### Clinical assessments

Assessment of motor function was performed with the UPDRS. General cognition was assessed with the Mini-Mental State Examination and Montreal Cognitive Assessment (MoCA). All participants had an MoCA score ≥26, above the cutoff for Parkinson dementia of the Movement Disorder Society Task Force criteria. Mild cognitive impairment (MCI) was defined as impaired performance on at least 2 neuropsychological tests according to Movement Disorder Society criteria. All participants underwent ophthalmology assessment, including optical coherence tomography, by an ophthalmologist. Participants with significant ophthalmic disease that could lead to concurrent Charles Bonnet syndrome and those with other neurologic disorders were excluded. Visual acuity was evaluated with LogMAR. Color vision was assessed with the D15 and contrast sensitivity with the Pelli-Robson test. Sniffin' Sticks were used to test olfaction. Mood was assessed with the Hospital Anxiety and Depression Scale and sleep with the REM Sleep Behavior Disorder Questionnaire. Levodopa dose equivalence scores were calculated for participants with PD.

### MRI data acquisition

All MRI data were acquired on a 3T Siemens Magnetom Prisma scanner (Siemens, Munich, Germany) with a 64-channel head coil. DWI was acquired with the following parameters: b = 50 s/mm^2^/17 directions, b = 300 s/mm^2^/8 directions, b = 1,000 s/mm^2^/64 directions, b = 2,000 s/mm^2^/64 directions, 2 × 2 × 2–mm isotropic voxels, echo time 3,260 milliseconds, repetition time 58 milliseconds, 72 slices, 2-mm thickness, and acceleration factor of 2. Acquisition time for DWI was ≈10 minutes. A 3D magnetization-prepared rapid-acquisition gradient echo image (voxel size 1 × 1 × 1 mm, echo time 3.34 milliseconds, repetition time 2,530 milliseconds, flip angle 7°) was also obtained and was used to compute intracranial volume with SPM12.

### DWI prepossessing

DWIs underwent denoising,^17^ removal of Gibbs ringing artifacts,^18^ eddy-current and motion correction,^19^ and bias field correction.^20^ To increase anatomic contrast and to improve statistics, we upsampled the DWI spatial resolution of DWIs to a voxel size of 1.3 mm^3^ and performed intensity normalization across participants.^21^ All preprocessing steps were performed with MRtrix3 (mrtrix.org).

Next, we calculated fiber orientation distributions (FODs) for each participant via Multi-Shell Multi-Tissue Constrained Spherical Deconvolution using the group average response function for each of the 3 tissue types (gray matter, white matter, and CSF).^22^ An unbiased, study-specific FOD template was created using FOD images from 30 randomly selected participants (20 with PD: 10 low and 10 high visual performers, 10 healthy controls) in accordance with previous studies.^23^ Each participant’s FOD image was then registered to the template using FOD-guided nonlinear registration.^24^ A tractogram with 20 million streamlines was generated with whole-brain probabilistic tractography on the population FOD template. This was then filtered to 2 million streamlines using spherical-deconvolution informed filtering of tractograms.^25^

### Fixel-based analysis

Classic diffusion tensor imaging approaches have limited ability to model crossing fibers.^26^ This limits their fiber specificity, especially in regions containing crossing fibers, which includes up to 90% of the white matter voxels in the brain.^27^ Fixel-based analysis is an emerging framework that uses a higher-order diffusion model to estimate the orientation of each fiber population and to quantify degenerative changes in specific fiber populations within voxels. This allows comparisons of specific fiber populations within voxels (or fixels) instead of comparing measures averaged across voxels.^28^

Using fixel-based analysis allows the characterization of more specific white matter tracts and provides information on their morphology and density. Detailed methodology of fixel-based analysis and interpretation of the derived measures have been previously described.^23,28,29^ Three measures were derived for each participant:

1. Fiber density (FD) or apparent FD. FD is sensitive to alterations in within-voxel level. FD is calculated as the integral of the FOD of each fixel and is proportional to the intra-axonal volume of fiber bundles aligned with that fixel. The FD is therefore a measure of microstructural changes within the tracts.^21,29^
2. Fiber cross-section (FC). This is a relative metric of differences in bundle cross section. Values >1 compared to the template imply larger cross section in that participant, and lower values suggest atrophy of a tract.^28^ FC is calculated as the extent of distortion in bundle cross section that is required to warp a participant’s FOD into the template image. This metric is a measure of macrostructural white matter change; it provides morphologic information about relative sizes of fiber bundles.^28^
3. Combined measure of FD and FC (FDC). Changes to a tract may be both macrostructural, with reduced FC, and within voxel, with lower FD in that fiber. This is captured by the FDC metric, which is calculated as FD multiplied by FC for each fixel and represents a combined measure of change at both the microstructural and macrostructural levels or, in other words, an overall measure of ability to relay information.^28^

**Figure 1:**
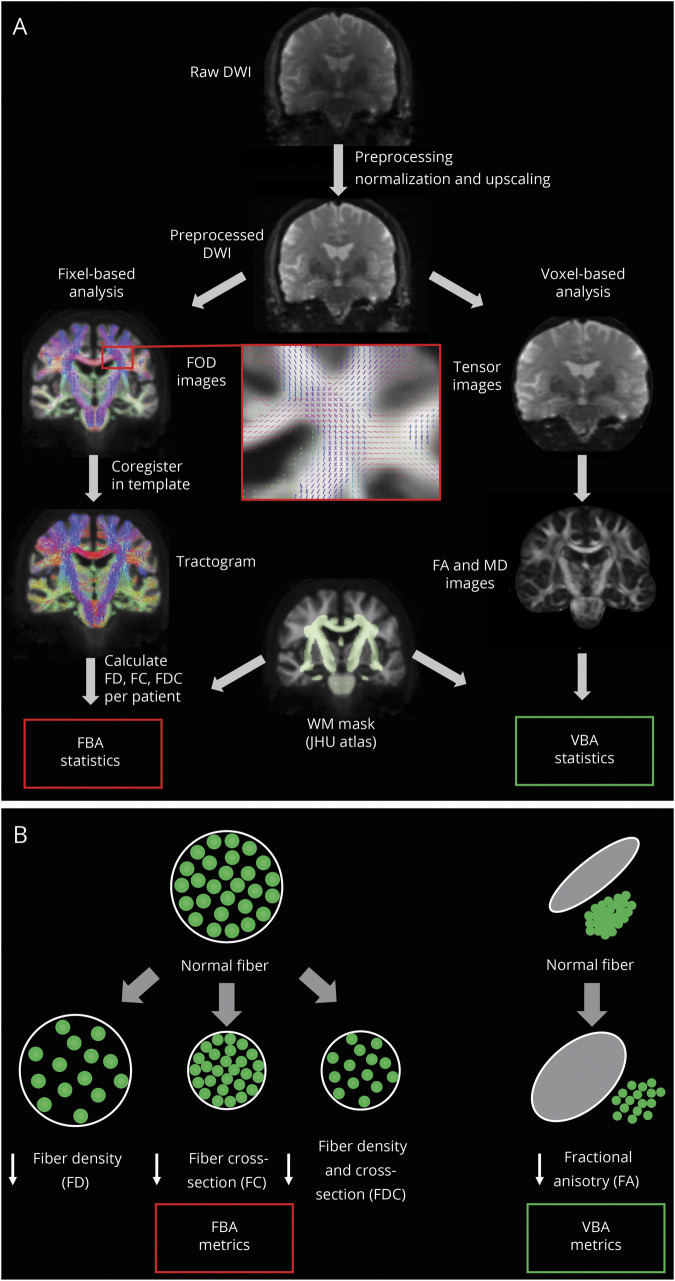
Overview of the processing steps involved in FBA and VBA whole-brain analyses (A) Processing steps for fixel-based analysis (FBA) and voxel-based analysis (VBA). Processing steps specific to FBA are shown on the left; those specific to VBA, on the right. (B) Illustration of the derived FBA and VBA metrics. DWI = diffusion-weighted imaging; FA = Fractional anisotropy; FC = fiber cross-section; FD = fiber density; FDC = fiber density and cross section combined; FOD = fiber orientation distribution; MD = mean diffusivity.

The processing steps for whole brain fixel-based analysis used in this study are shown in figure 1.

### Voxel-based analysis

To compare the results of fiber-specific fixel-based analysis with more commonly used measures of white matter integrity, we also performed a voxel-based analysis of fractional anisotropy (FA) and mean diffusivity (MD). Following the DWI preprocessing steps, we derived the diffusion tensor from each participant’s FOD image and calculated an FA and MD map in each participant’s space. Each individual participant’s FA and MD maps were then transformed to template space, using the same warp generated during the fixel-based analysis registration step. We then performed voxel-wise analysis in template space (figure 1).

### Statistical analysis

#### Demographic and clinical assessments

Differences in demographics and clinical characteristics between groups were examined with independent-sample *t* tests and analyses of variance for normally distributed continuous variables, Mann-Whitney and Kruskal-Wallis tests for nonnormally distributed ones, and χ^2^ for categorical variables. Visual inspection of variable distribution and the Shapiro-Wilk test were used to assess normality. Post hoc corrections for multiple comparisons were performed with the Tukey test for analysis of variance and the Nemeyni test for Kruskal-Wallis. Statistical significance was set at *p* < 0.05. All statistical analysis was performed in Python 3 with Jupyter Notebook version 5.5.0.

#### Whole-brain fixel-based analysis

We used whole-brain fixel-based analyses to identify regions with changes in FC, FD, and FDC between patients with PD and controls, patients with PD/VH and PD/non-VH, and patients with PD with high vs low visual performance. Additional comparisons of interest included Parkinson MCI vs Parkinson non-MCI and correlation with MoCA and 2 clinical dementia risk scores.^2,3^ Subgroup analysis in patients with PD and low visual performance included hallucinators vs nonhallucinators. Whole-brain fixel-based analysis refers to the comparison of all white matter fixels within the brain, as is standard for this analysis.^23^ Statistical comparisons of the 3 fixel-based analysis–derived measures between groups were performed at each fixel level with a general linear model with age and total intracranial volume included as nuisance covariates. We used connectivity-based fixel enhancement for statistical inference,^30^ using 2 million streamlines with default smoothing parameters (smoothing millimeter full width at half-maximum, C = 0.5, E = 2, H = 3), 5,000 permutations using nonparametric permutation testing, and family-wise error correction for multiple-comparison corrections; values of *p* < 0.05 were considered statistically significant.

#### Whole-brain voxel-based analysis

Voxel-based analysis was performed with threshold-free cluster enhancement with default parameters (dh = 0.1, E = 0.5, H = 2)^31^ across the whole brain as in the fixel-based analysis for the same comparisons: patients with PD vs controls, patients with PD/VH vs PD/non-VH, and patients with PD with high vs low visual performance. Age and total intracranial volume were included as nuisance covariates; family-wise error correction was used, and values of *p* < 0.05 were considered statistically significant.

#### Tract-of-interest analysis

To investigate potential degeneration of selective fiber pathways within the visual system, we also performed tract-of-interest analyses. On the basis of an a priori hypothesis that tracts from visual processing regions to the rest of the brain would be affected, we selected 11 white matter tracts involved in visual processing, using anatomic diffusion tensor imaging atlases,^32^ and computed the average FDC across the fixels of each defined tract. These tracts were the posterior thalamic and optic radiations, splenium, body and genu of the corpus callosum, superior longitudinal fasciculi, inferior fronto-occipital fasciculi (segmentation includes the inferior longitudinal fasciculus), and superior fronto-occipital fasciculi. Mean FDC was calculated across each tract per participant. FDC was chosen for tract-of-interest analysis because it is a combined metric of both microstructural and macrostructural changes representing the overall ability to relay information, and hence it is likely to be the most sensitive of the 3 fixel-based analysis–derived metrics. This is in accordance with other fixel-based analysis studies.^23^ Mean FDC was compared across groups: PD vs control, PD/VH vs PD/non-VH, and PD high performers vs PD low performers. Statistical comparison was performed with a linear mixed model (with age and intracranial volume as covariates) in Python 3. A false discovery rate (FDR) correction was performed for the 11 tracts tested using the Benjamini-Hochberg method. Correlational analyses of mean tract FDC with hallucination severity were performed with linear regression with age and total intracranial volume as covariates. A significance threshold of 0.05 was used. We display mean FDC results (and 95% confidence interval) as percentage difference from the mean FDC of the comparison group.

### Data availability

Anonymized data will be shared on request from any qualified investigator for purposes of replicating procedures and results.

## Results

### Demographic and clinical characteristics: VH vs non-VH

One hundred forty participants were included: 105 patients with PD and 35 controls. Nineteen patients described recurrent VH (PD/VH) and 86 did not (PD/non-VH). Participants with PD/VH and PD/non-VH did not significantly differ in demographics or low-level vision. Those with PD/VH had higher total UPDRS score (*p* = 0.003, table 1), but motor handicap was not significantly different between the 2 groups. Participants with PD/VH had higher anxiety scores than those with PD/non-VH (*p* = 0.037), but this was below the threshold for diagnosis of anxiety.^33^ Of note, age, sex, intracranial volume, cognition, and performance in the visual experimental tasks did not differ significantly between the groups (table 1).

**Table 1:**
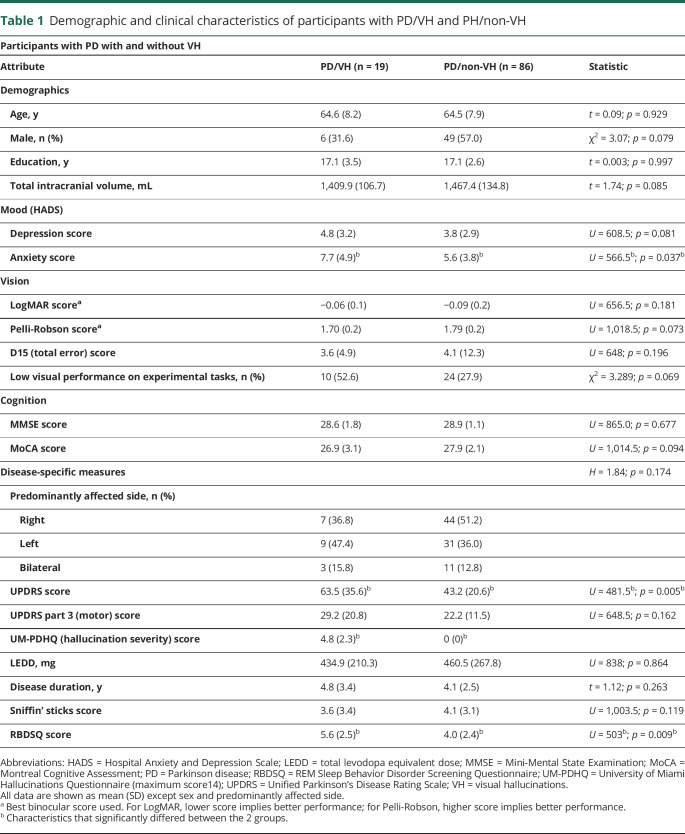
Demographic and clinical characteristics of participants with PD/VH and PH/non-VH

### Demographic and clinical characteristics: High vs low performers on visual testing

Thirty-four participants with PD were low performers and 70 were high performers on visual testing. Age, sex, intracranial volume, and cognitive measures did not differ between low and high performers with PD. Low performers with PD had worse contrast sensitivity (*p* < 0.001, table 2) and a higher propensity for hallucinations (*p* = 0.041), with 29.4% being habitual hallucinators (table 2). Depression scores were higher in the PD low performer group, but they were below the threshold for depression^33^ (table 2).

**Table 2:**
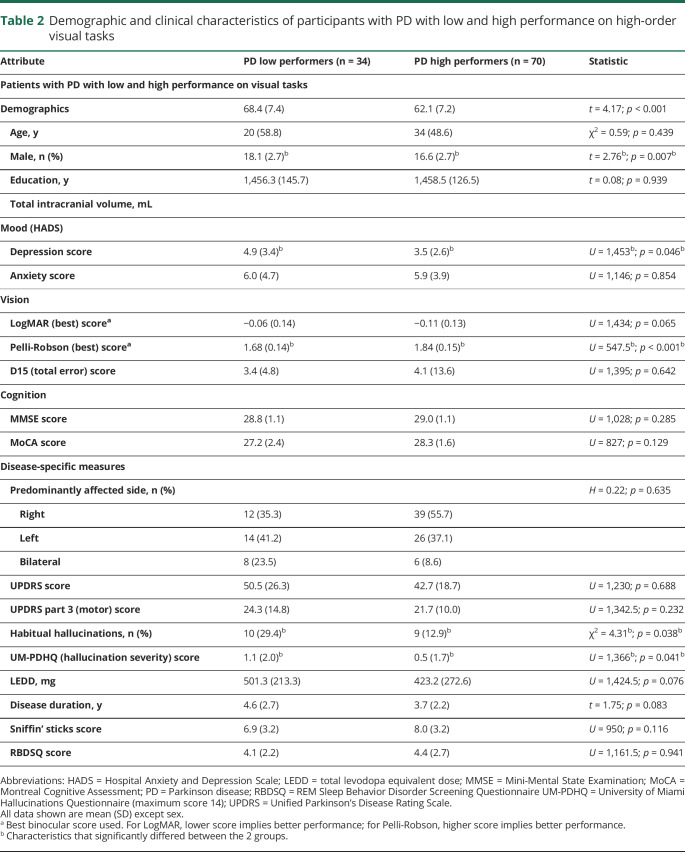
Demographic and clinical characteristics of participants with PD with low and high performance on high-order visual tasks

Demographic and clinical characteristics of the control participants are available at Figshare (figshare.com/s/1ff87201e92924f488f2).

### Fixel-based analysis

#### Patients with PD vs controls

Whole-brain fixel-based analysis comparing participants with PD and controls showed macrostructural white matter changes with reduced FC in multiple fixels within the left corticospinal tract (data available at Figshare, figshare.com/s/1ff87201e92924f488f2). There were no differences at the microstructural level, measured as FD or the combined metric FDC, between patients with PD and control participants. Within the PD group, overall disease severity, as assessed with the UPDRS total score, was associated with decreased FC within the splenium of the corpus callosum (data available at Figshare).

#### White matter atrophy in PD/VH

**Figure 2:**
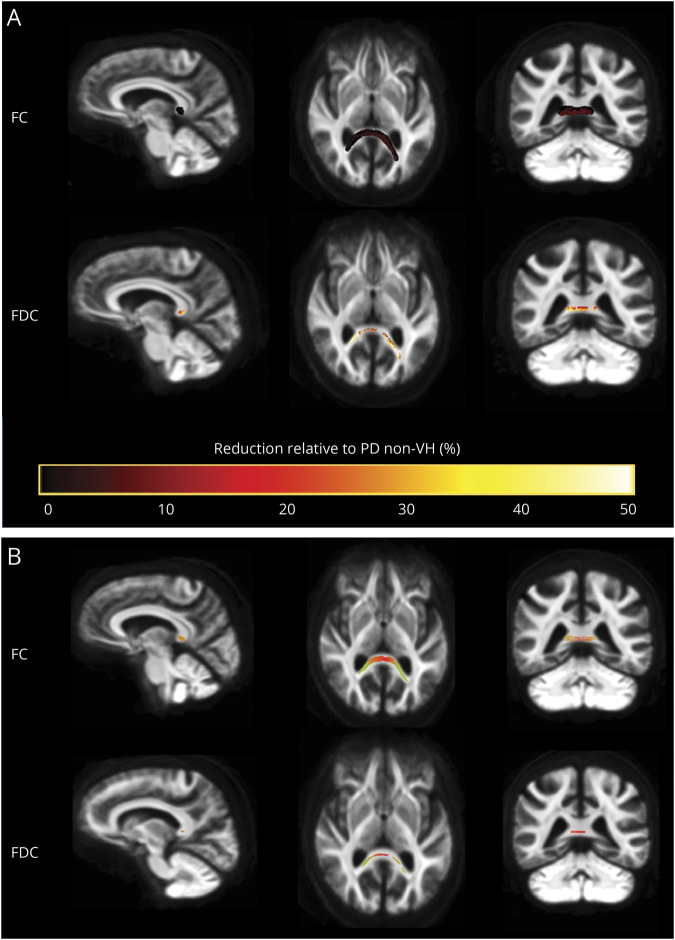
Fiber tract–specific reductions in PD/VH compared to PD/non-VH from whole-brain fixel-based analysis (A) Patients with Parkinson disease (PD) with visual hallucinations (VH) vs patients with PD/non-VH. Patients with PD/VH showed changes in white matter macrostructure (as seen by reduction in fiber cross section [FC]) and the overall ability to relay information (as defined by reduction in the combined FC/fiber density [FDC] metric) compared to patients with PD/non-VH. Reduced FC and FDC are seen in the splenium of the corpus callosum and the left optic radiation in PD/VH. Percentage reduction in FC and FDC is shown (color bar reflects percentage reduction) (family-wise error [FWE]–corrected *p* < 0.05). (B**)** PD/VH vs PD/non-VH, direction of fibers. Loss of fiber tracts in PD/VH compared with PD/non-VH was seen particularly for left-right axons (color bar reflects direction of fiber loss: anterior-posterior, green; superior-inferior, blue; left-right, red) (FWE-corrected *p* < 0.05).

Patients with PD/VH showed white matter macrostructural changes, with reductions in FC within the splenium of the corpus callosum and the left optic radiation, compared with nonhallucinators. The combined FDC metric, which assesses microstructural and macrostructural white matter changes, showed large reductions (>50% reduction) in participants with PD/VH compared with PD/non-VH within the splenium (figure 2). There were no significant changes in FD alone.

#### Changes in white matter microstructure and morphology related to low visual performance in PD

**Figure 3:**
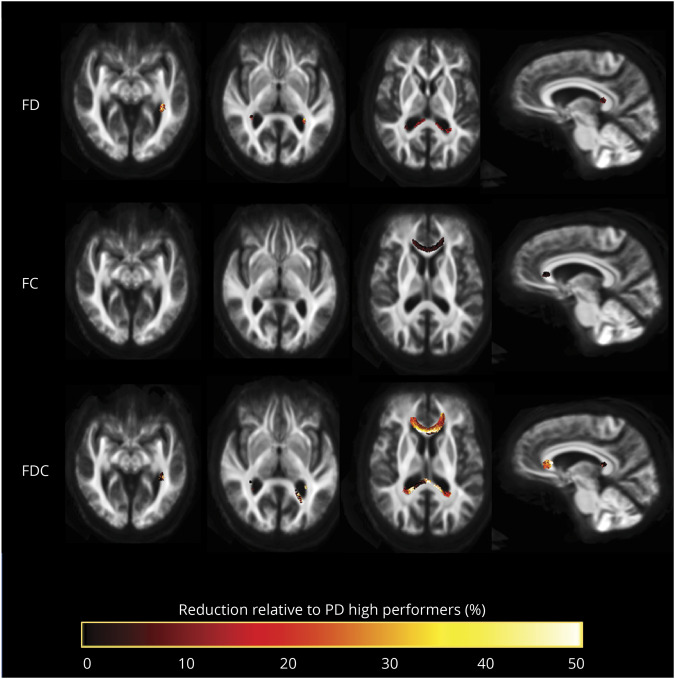
Fiber tract-specific reductions in PD low performers compared to PD high performers from whole-brain fixel-based analysis Patients with Parkinson disease (PD) and low visual performance showed macrostructural (changes in fiber cross section [FC]) and microstructural changes (changes in fiber density [FD]). Microstructural changes are seen within the splenium of the corpus callosum, posterior thalamic radiations bilaterally, and left inferior fronto-occipital fasciculus. Macrostructural changes are seen within the corpus callosum. Changes in the combined FD/FC (FDC) metric are seen in the genu, splenium, bilateral thalamic radiations, and left inferior fronto-occipital fasciculus; this represents impaired overall ability to relay information in these tracts in PD low performers. Results are displayed as streamlines; these correspond to fixels that significantly differed between PD low performers and PD high performers (family-wise error–corrected *p* < 0.05). Streamlines are colored by percentage reduction in the PD low performers group compared to high performers for FD, FC, and FDC.

Patients with PD and low visual performance showed significant microstructural changes, with reductions in FD in multiple white matter tracts. Specifically, reductions were seen within the splenium of the corpus callosum, posterior thalamic and optic radiations bilaterally, and left inferior fronto-occipital fasciculus, with peak reductions (greater than 50% reductions) in the left inferior fronto-occipital fasciculus. Macrostructural changes, measured with FC, were found within the genu of the corpus callosum. The combined FDC metric showed significant reductions in the genu, splenium, bilateral thalamic radiations, and left inferior fronto-occipital fasciculus in low performers compared to high performers with PD (figure 3). They were particularly pronounced within the corpus callosum (genu and splenium) with >50% reductions in FDC.

Subgroup analysis within the group of PD low performers was performed to assess whether the findings were driven by the hallucinator subgroup. We found no significant differences in FC, FD, or FDC between PD low performers with hallucinations and without hallucinations.

White matter changes were not seen for measures of general cognition or dementia risk. MoCA, status of MCI, and 2 clinical dementia risk scores^2,3^ were not associated with any significant white matter changes at the microstructural or macrostructural level, suggesting that visual performance is more strongly linked to structural changes in the predementia state in PD.

### Voxel-based analysis

Conventional voxel-based analysis of FA and MD did not show any statistically significant differences between any of the tested groups: PD vs controls, PD high performers vs PD low performers, and PD/VH vs PD non VH.

### Tract-of-interest analysis

#### FDC changes in patients with PD

In tract-of-interest analysis, we saw increased mean FDC in patients with PD compared to controls in the genu of the corpus callosum (*t* = 3.135, *p* = 0.002) and the left inferior fronto-occipital fasciculus (*t* = 2.336, *p* = 0.019). The genu survived FDR correction for multiple comparisons (*p* = 0.016). There were no significant differences between patients with PD and controls in the remaining 9 tracts.

#### FDC changes associated with VH

**Figure 4:**
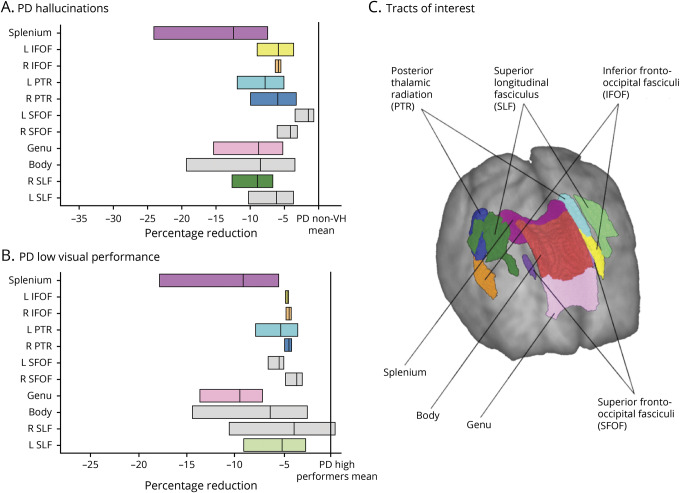
Significant tracts in patients with PD/VH and PD low performers; tract-of-interest analysis (A) Reduction (mean, 95% confidence interval [CI]) in combined fiber density and cross-section (FDC) visualized as percentage reduction from the mean of patients with Parkinson disease (PD) without hallucinations (PD/non-VH). The posterior thalamic radiations bilaterally, inferior fronto-occipital fasciculi bilaterally, genu, and right superior longitudinal fasciculi survived false discovery rate (FDR) correction. (B) Reduction (mean, 95% CI) in FDC visualized as percentage reduction from the mean of patients with PD with high visual performance. Note that only the genu survived FDR correction. Tracts with significantly reduced FDC (*p* < 0.05) are shown in color; tracts where there are no significant changes in FDC are plotted in gray. (C) Anatomic representation of all analyzed tracts.

We saw widespread reductions in mean FDC in PD/VH compared to PD/non-VH in a tract-of-interest analysis. Specifically, changes were seen in the splenium, the inferior fronto-occipital fasciculus bilaterally, the posterior thalamic radiation bilaterally, and the superior longitudinal fasciculus on the right (table 3). The inferior fronto-occipital fasciculi bilaterally survived FDR correction for the regions tested (right *p* = 0.033, left *p* = 0.033), as did both posterior thalamic radiations (right *p* = 0.033, left *p* = 0.037), the genu (*p* = 0.037), and the right superior longitudinal fasciculus (*p* = 0.033) (figure 4). Including cognition (MoCA score) as a covariate in the analysis did not influence results, indicating that the observed white matter changes are independent of cognitive performance.

**Table 3:**
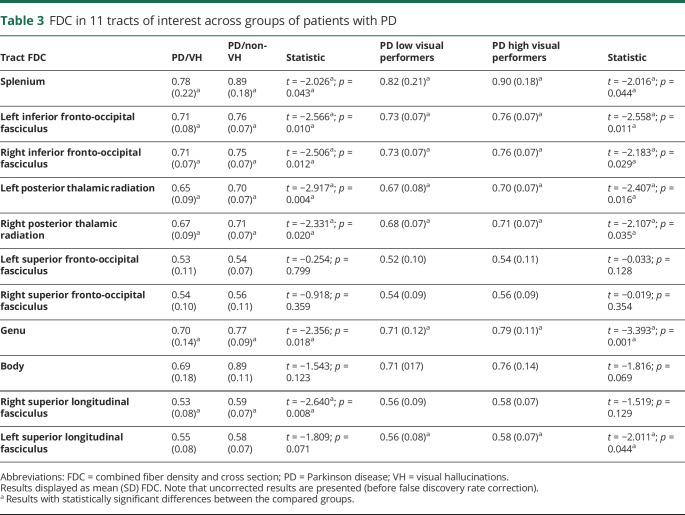
FDC in 11 tracts of interest across groups of patients with PD

Higher hallucination severity, determined by University of Miami Hallucinations Questionnaire, was associated with lower mean FDC within the left posterior thalamic radiation, accounting for age and total intracranial volume, although this was just above statistical significance levels (*r*^2^ = 0.316, *df* = 101, *t* = −1.955, *p* = 0.053).

#### FDC changes in PD low performers

There were widespread reductions in mean FDC in PD low visual performers compared to PD high performers across the visual system. Specifically, FDC reductions were seen in the splenium, the genu, the inferior fronto-occipital fasciculi bilaterally, the posterior thalamic radiations bilaterally, and the left superior longitudinal fasciculus (table 3), with the genu surviving FDR correction for the regions tested (*p* = 0.011) (figure 4). Again, controlling for MoCA scores had no influence on the reported results.

## Discussion

Here, we have used fixel-based analysis to measure fiber-specific changes in patients with PD/VH and those with poor visual function to shed light on the early stages of cognitive impairment in PD. We found that patients with PD/VH exhibited axonal reduction within the splenium and left posterior thalamic radiation and that low visual performance in PD was associated with changes in the splenium and genu of the corpus callosum, posterior thalamic radiations, and left inferior fronto-occipital fasciculus. These findings provide evidence that visual changes in PD are associated with microstructural and microstructural changes within specific fiber pathways that have been functionally implicated in PD dementia.

Our finding of white matter changes in posterior thalamic projections is consistent with previous work using tensor-derived metrics that showed reduced FA in the optic nerve and optic radiation in an analysis of 5 manually determined regions of interest^34^ and increased MD within tracts from the nucleus basalis of Meynert to parietal and occipital regions in patients with PD/VH.^35^ In contrast, past whole-brain tensor-based analyses had not revealed any specific white matter changes between PD/VH and PD/non-VH.^36,37^ We found widespread reductions in FC, as well as the FDC metric, suggesting that white matter macrostructural changes occur in Parkinson hallucinations together with changes that affect the ability to relay information between brain regions.

The posterior thalamic radiation connects the posterior thalamus with the occipital and posterior parietal cortex, including key regions of the default mode network (DMN) such as the posterior cingulate. Several functional studies have highlighted the role of attentional networks in the development of VH.^38,39^ Our finding of reduced connectivity from the thalamus to posterior brain regions, including the DMN, provides a mechanistic model whereby lack of inhibition between these brain regions leads to aberrant DMN activation.^38,39^

In tract-of-interest analysis, we also saw FDC reductions within association fibers in PD hallucinators, specifically those connecting frontal and occipital lobes (inferior fronto-occipital fasciculi bilaterally and right superior longitudinal fasciculus). This highlights the importance of loss of fronto-occipital connectivity in the development of hallucinations.

The patients with PD/VH had higher anxiety and REM Sleep Behavior Disorder Questionnaire scores than the nonhallucinators. However, anxiety scores were below the clinical significant threshold for depression,^33^ and the small observed changes in anxiety and RBD are unlikely to have resulted in white matter changes within tracts of the visual system.

We found widespread changes in FD in patients with PD and poor visual function, particularly in the splenium, bilateral posterior thalamic projections, and left inferior fronto-occipital fasciculus. This is in comparison to a reduction in FC, reflecting fiber atrophy, that was restricted to the genu of the corpus callosum. The most pronounced changes in patients with PD and low visual performance, in both spread and effect, were seen in the combined FDC metric. This overall connectivity metric combines density and morphology information, is thought to reflect differences in the ability to relay information between brain regions,^28^ and was most reduced within the genu in PD low performers in whole-brain and tract-of-interest analysis. Disconnection of frontal interhemispheric processing may be linked to poor visual performance and has previously been implicated in worse visual search performance in healthy aging.^40^ In tract-of-interest analysis, the genu of the corpus callosum showed increased mean FDC in patients with PD compared to age-matched controls; this could be a result of heterogeneity within patients with PD.

Changes within the corpus callosum have previously been described using tensor-derived metrics in patients with PD and cognitive impairment in both whole-brain and region-of-interest analyses, with changes being particularly pronounced in the most anterior and most posterior segments.^13,14,41^ We also found significant microstructural and macrostructural changes within the genu and the splenium of the corpus callosum in patients with PD and low visual performance. There is growing evidence that visuoperceptual deficits are an early indicator of PD dementia,^1^ and we have previously shown that visuoperceptual deficits are linked to cognitive decline after 1 year.^6^ By examining the differences in white matter connectivity in patients with PD and low visual performance and using a novel, more sensitive technique, we have highlighted additional white matter tracts that are affected in the earliest stages of cognitive impairment.

Our results are also in keeping with other neuroimaging studies highlighting the importance of connectivity changes in early cognitive impairment of PD. Changes in functional connectivity between visuospatial and frontal regions^42^ and between the posterior cingulate with frontal regions^6,42^ are seen in patients with PD and early cognitive impairment. We have also shown changes within the left fronto-occipital fasciculus, an association tract thought to play a role in executive function, semantic language, and visual processing.^43^ Measures of global connectivity are also reduced in patients with PD and cognitive impairment compared to those without in both functional and structural imaging data.^44,45^ Diffuse changes in white matter tracts in patients with PD and poor visual performance were also demonstrated in our study, consistent with reduction in global connectivity.

Subgroup analysis within patients with PD with low visual performance did not show any significant differences between those with low visual performance and hallucinations and those with low visual performance without hallucinations. This indicates that the affected white matter tracts are associated only with poor visual performance and are not driven by the hallucinator subgroup. In addition, clinical risk scores for dementia, MCI status, and MoCA were not associated with white matter loss, suggesting that measures of high-level visual function may be more sensitive than standard global measures to detect early cognitive change in PD.

No significant changes were seen in FA or MD between patients with PD with low vs high visual performance or in hallucinators vs nonhallucinators. Because tensor-based metrics are, by definition, averaged across a voxel, their results can be misleading, particularly in the presence of crossing fibers.^26^ In contrast, our fixel-based analyses identified group differences in fiber microstructure and macrostructure. This suggests that high-order diffusion models and specifically fixel-based analysis may be more sensitive and specific for examining white matter changes in the early stages of neurodegeneration.

The white matter tracts that were most affected in the PD low performers included areas that connect key regions of the DMN. The genu of the corpus callosum contributes to homotopic connections between prefrontal brain regions. Thus structural changes within the genu could lead to functional disconnection of the anterior medial prefrontal cortex.^46^ Similarly, the splenium connects association areas of the posterior inferior parietal, temporal, and occipital lobes.^46^ The inferior fronto-occipital fasciculus connects medial and inferior frontal gyri to occipital gyri but also angular, fusiform, and lingual gyri and the cuneus.^43^ Although observed functional connectivity is not fully explained by direct structural white matter connectivity, changes within anatomic white matter tracts could predict resulting changes in functional connectivity.^47^ Our findings are consistent with previous studies using functional metrics that have suggested that DMN activation is reduced in PD, with loss of connectivity correlated with worse cognition.^48^

There are a number of potential limitations to our study. We did not exclude participants with other pathologies that could influence white matter structure such as white matter hyperintensities, which could decrease FD. Although raw imaging data were visually inspected and no clinically significant cerebrovascular disease was seen, the load of white matter hyperintensities was not systemically quantified or specifically controlled for. This is in keeping with other studies of fixel-based analysis in adults so far, none of which excluded participants with white matter hyperintensities.^23,49^ Future studies could clarify the effect, if any, of white matter hyperintensities on fixel-based metrics.

Our participants underwent imaging acquisition while taking their usual dopaminergic medications. It seems unlikely that dopaminergic medications would directly affect structural integrity measures, and free water and corrected FA are not affected by levodopa medication.^50^

Finally, the current study examines cross-sectional data, and longitudinal studies examining white matter changes in patients with visual deficits who progress to dementia are likely to provide useful insights into the earliest fibers affected in patients with PD who go on to develop dementia.

We show that patients with PD/VH have a characteristic pattern of fiber tract degeneration, involving the splenium and posterior thalamic radiation, and that patients with PD and poor visual function show widespread changes in FD, especially in the splenium, posterior thalamic radiations, genu, and left inferior fronto-occipital fasciculus. These findings provide mechanistic support for attentional network changes in Parkinson hallucinations and insights into early stages of cognitive involvement in PD.

## Glossary

DMN: default mode network
DWI: diffusion-weighted imaging
FA: fractional anisotropy
FC: fiber cross section
FD: fiber density
FDC: combined FD and FC
FDR: false discovery rate
FOD: fiber orientation distribution
MCI: mild cognitive impairment
MD: mean diffusivity
MoCA: Montreal Cognitive Assessment
PD: Parkinson disease
RBDSQ: REM Sleep Behavior Disorder Questionnaire
UPDRS: Unified Parkinson's Disease Rating Scale
VH: visual hallucinations
